# Co-benefits of CO_2_ emission reduction from China’s clean air actions between 2013-2020

**DOI:** 10.1038/s41467-022-32656-8

**Published:** 2022-08-27

**Authors:** Qinren Shi, Bo Zheng, Yixuan Zheng, Dan Tong, Yang Liu, Hanchen Ma, Chaopeng Hong, Guannan Geng, Dabo Guan, Kebin He, Qiang Zhang

**Affiliations:** 1 School of Environment, Tsinghua University, Beijing, China; 2 Department of Earth System Science, Tsinghua University, Beijing, China; 3 Institute of Environment and Ecology, Tsinghua Shenzhen International Graduate School, Tsinghua University, Shenzhen, China; 4 Center of Air Quality Simulation and System Analysis, Chinese Academy of Environmental Planning, Beijing, China; 5 Institute for Carbon Neutrality, Tsinghua University, Beijing, China

**Keywords:** Environmental impact, Climate-change policy

## Abstract

Climate change mitigation measures can yield substantial air quality improvements while emerging clean air measures in developing countries can also lead to CO_2_ emission mitigation co-benefits by affecting the local energy system. Here, we evaluate the effect of China’s stringent clean air actions on its energy use and CO_2_ emissions from 2013-2020. We find that widespread phase-out and upgrades of outdated, polluting, and inefficient combustion facilities during clean air actions have promoted the transformation of the country’s energy system. The co-benefits of China’s clean air measures far outweigh the additional CO_2_ emissions of end-of-pipe devices, realizing a net accumulative reduction of 2.43 Gt CO_2_ from 2013-2020, exceeding the accumulated CO_2_ emission increase in China (2.03 Gt CO_2_) during the same period. Our study indicates that China’s efforts to tackle air pollution induce considerable climate benefit, and measures with remarkable CO_2_ reduction co-benefits deserve further attention in future policy design.

## Abstract (web summary)

China’s clean air action stimulated a net accumulative reduction of 2.43 Gt CO_2_ emission from 2013-2020. Phase-out and upgrades of outdated, polluting, and inefficient combustion facilities have promoted the transition of the country’s energy system.

## Introduction

With rapid economic development and urbanization, China has become the largest energy consumer in the world. The massive consumption of fossil fuels has resulted in severe air pollution and CO_2_ emissions growth. To improve air quality, the Chinese government released the Air Pollution Prevention and Control Action Plan (the Action Plan) in 2013, aiming to achieve a notable decrease in the fine particulate (PM_2.5_) concentration in key regions by the end of 2017. It is followed by the Three-Year Action Plan for Winning the Blue Sky Defense Battle in 2018, the second phase of the Action Plan, that required further nationwide air quality improvements. Air quality in China was dramatically improved after eight years of efforts, driven by substantial declines in air pollutant emissions^1,2^.

Clean air measures can also promote CO_2_ emission mitigation by affecting local energy systems, although the original policy objective is not related to climate change^3–5^. Energy-related measures in China’s clean air actions include energy use caps, energy structure adjustments, and energy efficiency improvements, which lead to a reduced fossil fuel consumption and co-benefits of CO_2_ emission reduction. After decades of explosive growth, China’s CO_2_ emissions surprisingly entered a four-year plateau around 2013^6–10^. Current understanding of how clean air policies affect climate change focuses on the air pollution-induced climate forcing^11–15^, while the effects on reducing CO_2_ are poorly understood. Previous regional-focused studies have demonstrated CO_2_ emission reduction co-benefits of implemented clean air action measures^16,17^. However, how the different air pollution control measures in China’s two-phase clean air actions stimulated CO_2_ emission reduction remains unclear, and the contributions of China’s Clean Air Action to the deceleration in the growth of China’s CO_2_ emissions since 2013 have not been quantified.

In this work, the CO_2_ emission reduction co-benefits of China’s clean air actions from 2013 to 2020 are quantified with the use of a modeling framework to assess the effectiveness of clean air measures on air quality improvements^1^. Based on a thorough review of China’s clean air actions, we summarize six measures activated or strengthened by China’s Clean Air Actions and distinguish the end-of-pipe measures with the other actions that alter the energy use efficiencies. Then, we carry out an ex-post assessment to quantify the impact of those energy-related measures on China’s energy end-use flow and CO_2_ emissions according to the real implementation rate of each measure collected by the government (unpublished data). CO_2_ emission reduction co-benefits of each measure were estimated by using the model of the Multi-resolution Emission Inventory of China (MEIC)^2,18–22^. Details are described in the “Methods” section.

## Results

### Trends in emissions and air quality from 2013 to 2020

**Fig. 1:**
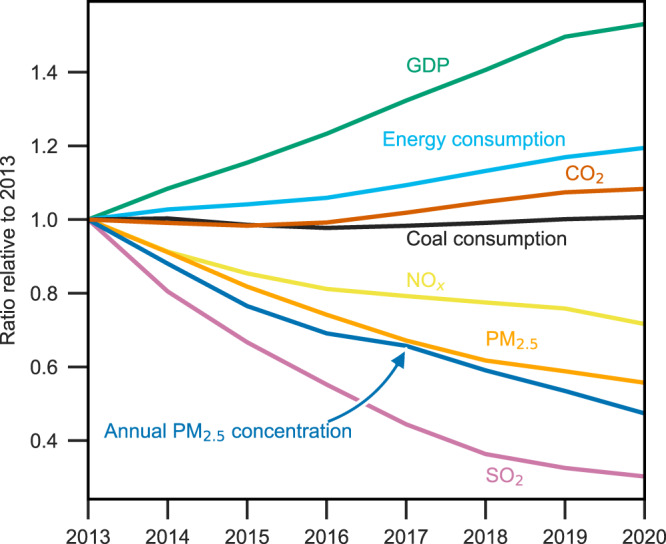
Trends of anthropogenic emissions, annual PM_2.5_ concentration, energy consumption and GDP in China between 2013 and 2020. The annual PM_2.5_ concentration was calculated based on ground-based observations data in 74 key cities.

China’s anthropogenic emissions of major air pollutants and CO_2_ from 2013 to 2020 were estimated through a bottom-up approach under the framework of the MEIC model. As shown in Fig. 1, emissions of SO_2_, NO_*x*_, and primary PM_2.5_ were estimated to decline by 69%, 28%, and 44%, respectively between 2013 and 2020. As the consequence, annual average concentration of PM_2.5_ in 74 key Chinese cities decreased from 72 μg m^−3^ in 2013 to 34 μg m^−3^ in 2020^23^. Although meteorological condition variations could also contribute to the changes in PM_2.5_ concentration, remarkable emission mitigation has been identified as the major driver of national air quality improvements during the clean air action period^1,24^. Sustained gross domestic product (GDP) growth and a notably improved air quality may indicate a decoupling of economic growth and air pollution in China^25^.

In contrast to the downward trends of air pollutant emissions, China’s energy consumption and CO_2_ emissions exhibited an overall upward trend during the same period. Following a rapid increase after 2000^26^, China’s CO_2_ emissions reached a plateau from 2013 to 2016, which is closely related to the downward trend of coal consumption. A rebound of CO_2_ emissions occurred after 2016 due to the growth of fossil fuel consumption^27–29^, which was dominated by the power sector. Thermal power generation in 2017 increased by 7.2% over the year of 2016, leading to a 220 Mt increase in CO_2_ emissions in the power sector. Despite the influence of the COVID-19 pandemic, China’s CO_2_ emissions did not exhibit a notable decline in 2020 as a result of effective COVID-19 control measures and the rapid recovery of economic activities after the lockdown^30^.

### Measure-specific CO_2_ reduction co-benefits

**Fig. 2:**
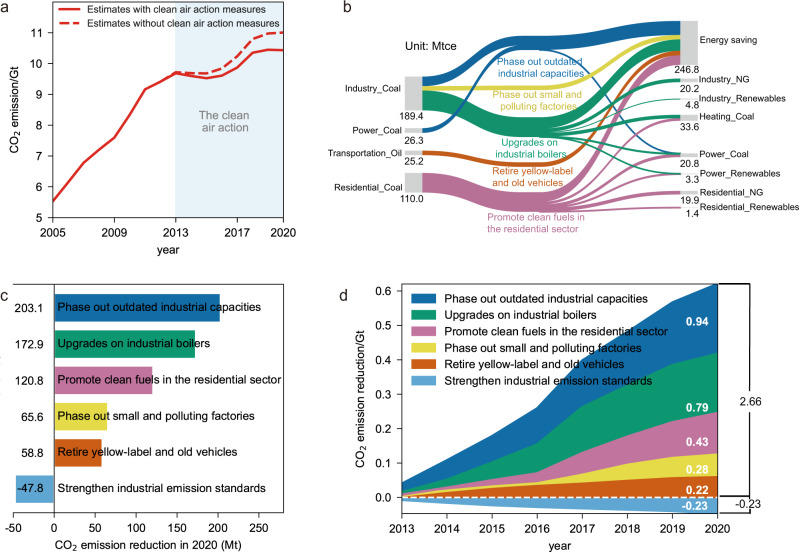
Change in China’s energy end-use flows and measure-specific contributions to CO_2_ emission reduction. **a** CO_2_ emission estimates with and without clean air action measures. **b** Transition in energy structure in China between 2013 and 2020. The transition of energy end-use flows was driven by five co-beneficial measures. The left side represents the reduction of energy consumption in 2020, and the right side represents the attribution of reduced energy consumption to energy savings and transformation into other cleaner energy sources. **c** Measure-specific CO_2_ emission reductions in 2020. Negative reduction from the enhancement of industrial emission standards represents the additional CO_2_ emissions due to application of end-of-pipe technologies. **d** Accumulated CO_2_ emission reduction between 2013 and 2020 by each measure. Numbers within graphs show total amounts of CO_2_ emissions reduction over the period.

By reviewing national and regional policy packages, we summarized the five major clean air measures which may also lead to co-reductions on CO_2_ emissions, i.e., upgrades on industrial boilers, phasing out small and polluting factories, phasing out outdated industrial capacity, promoting clean fuels in the residential sector, and retiring yellow-label and old vehicles. We then estimated the changes of energy use and CO_2_ emissions induced by each measure (see Methods). As the end-of-pipe emission control measures may use additional energy and increase CO_2_ emissions, we also quantified the CO_2_ emission increase as the result of strengthening industrial emission standards. According to our estimates, the implementation of China’s Clean Air Action successfully avoided 0.57 Gt anthropogenic CO_2_ emissions, which is 5.5% of the real-world emissions in 2020 (Fig. 2a). Figure 2b shows the energy end-use flows driven by five co-beneficial measures in 2020. Between 2013 and 2020, small, outdated combustion facilities were widely replaced by larger, cleaner, and more efficient infrastructures, which improved combustion efficiency and reduced energy use, especially coal use. As a result, the five co-beneficial measures provided a net energy savings of 0.25 giga tons coal equivalent (Gtce) in 2020 and accumulatively saved 1.06 Gtce energy between 2013 and 2020.

Figure 2c shows the subsequent CO_2_ emission reduction of each measure in 2020. The two most effective measures to reduce CO_2_ emissions include phasing out outdated industrial capacities and upgrading industrial boilers (Fig. 2c), which were estimated to reduce 0.20 and 0.17 Gt CO_2_ emissions, respectively, in 2020. Since 2013, outdated capacity in several industrial sectors were phased out and replaced with of advanced technologies, including a total coal-fired power plant production capacity of 45 GW, steel production capacity of 312 million tons, cement production capacity of 388 million tons and flat glass production capacity of 192 million weight boxes. Small industrial boilers with a capacity lower than 7 MW generally attain a low combustion efficiency as low as 65% and typically lack end-of-pipe pollution control. About 424 GW small coal-fired boilers were eliminated, replaced by more efficient boilers (with an efficiency as high as 84%), or were shifted to low-carbon energy sources, such as natural gas and biomass fuels.

Promoting clean fuels in the residential sector contributed 0.12 Gt CO_2_ emission reduction in 2020. Over 29 million households quit from coal-fired heating systems and shifted to natural gas and electricity under the clean air action, with more than 7.5 GW of residential coal-fired boilers eliminated. Phasing out small and polluting factories reduced emissions by another 0.07 Gt CO_2_. Starting in 2016, approximately 660 thousand small and polluting factories were shut down or upgraded, including factories manufacturing brick, lime, nonferrous metals, foundry products, and other industrial products. From 2017 to 2020, over 30 million tons of scattered coal use in small industrial furnaces were eliminated due to special rectification measures. The retirement of yellow-label and old vehicles contributed 0.06 Gt to CO_2_ reduction. More than 26 million yellow-label and old vehicles (i.e., gasoline vehicles that do not meet the China III emission standards and diesel vehicles that do not meet the China IV emission standards) were early retired between 2013 and 2020. On the other hand, we estimated that installing end-of-pipe control equipment have increased 0.05 Gt CO_2_ in 2020, with major contribution from the power sector and the iron and steel sector (Supplementary Table 1).

Our estimates suggest that China’s clean air measures are associated with a cumulative CO_2_ emission reduction of 2.66 Gt between 2013 and 2020, much larger than the cumulative CO_2_ emission increase of 0.23 Gt from newly installed end-of-pipe pollution control devices (Fig. 2d). The net cumulative emission reduction reached 2.43 Gt, 3.1% of China’s CO_2_ emissions between 2013 and 2020. It also surpassed the accumulated CO_2_ emission increases in China (2.03 Gt CO_2_) during the same period. The net emission reduction gradually increased from 2013 to 2017 due to the accelerated adjustment of energy and industrial structures. For instance, the energy savings from industrial kilns/furnaces and the residential sector rose sharply in 2017, twice as fast as those in 2016, as the government aimed to achieve its goal in the final year of the first phase of the Action Plan. The clean air measures after 2017 focused more on industrial emissions reduction through highly efficient end-of-pipe technologies and the control of diffusive emissions such as volatile organic compounds. A lower intensity of co-beneficial policy implementation resulted in a decelerated growth of CO_2_ emissions reduction co-benefits after 2017.

### Regional patterns

**Fig. 3:**
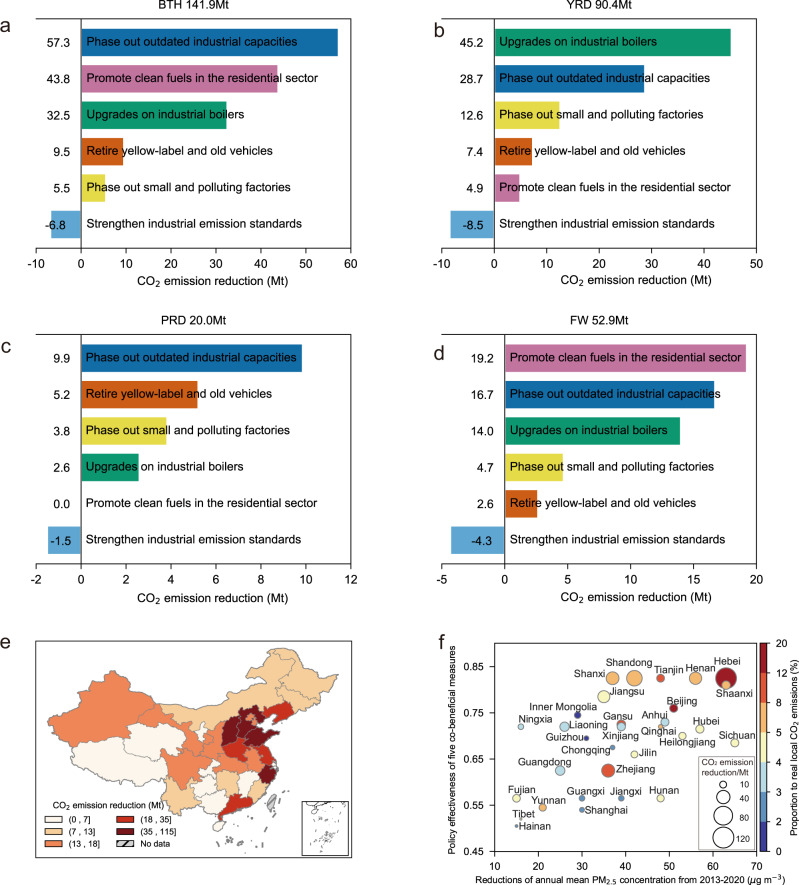
Regional patterns of CO_2_ emission reduction co-benefits in 2020. **a–d** CO_2_ emission reduction from six measures in Beijing–Tianjin–Hebei region (BTH), Yangtze River Delta region (YRD), Pearl River Delta region (PRD), and Fenwei Plain region (FW), respectively. **e** The provincial CO_2_ emission reduction in 2020. **f** The relationship between the policy effectiveness, air quality improvement, and CO_2_ emission reduction. Each bubble in **f** indicates a province, with the size representing the CO_2_ emission reduction in 2020 and the color representing the proportion of CO_2_ emission reduction to real local CO_2_ emissions in 2020. The policy effectiveness provides a general measure of the policy intensity in a region, with the coefficient value ranging from 0 (very weak) to 1 (very strong), and more details are presented in Supplementary Note 1 and Supplementary Tables 2 and 3.

The co-benefits of CO_2_ emission reduction from air pollution control measures are further analyzed at the regional level and presented in Fig. 3. The Beijing–Tianjin–Hebei region (BTH), Yangtze River Delta region (YRD), Pearl River Delta region (PRD) and Fenwei Plain region (FW) are four key air pollution control regions, accounting for 53.2% of China’s net co-benefits of CO_2_ emission reduction in 2020. BTH is the largest contributor, followed by YRD, FW, and PRD. The provinces included in these key areas are mainly located in eastern China, with a relatively advanced economy, large population, and high PM_2.5_ concentration.

Phasing out of outdated industrial capacity led to a considerable CO_2_ emission reduction in all key regions, but the fraction of emission reduction introduced by each measure revealed spatial disparity given the differences in emission patterns (Supplementary Fig. 1) and policy focuses. The most effective co-beneficial measures in BTH include outdated industrial capacity phase-out, clean fuel promotion in the residential sector, and industrial boiler upgrading, reflecting efforts to adjust the industrial structure and reduce scattered coal use. In YRD, industrial boiler upgrading was the most effective CO_2_ reduction policy. The co-benefits of phasing out small and polluting factories were also notable in YRD, reducing CO_2_ emissions by 12.6 Mt in 2020. While the increase in CO_2_ emissions originating from strengthening industrial emission standards in YRD reached 8.5 Mt CO_2_ emissions in 2020, which was primarily attributed to the wider application of end-of-pipe technologies in the power sector. CO_2_ emission reduction co-benefits in PRD were the lowest among the four regions, which may be attributed to its relatively good air quality and low CO_2_ emissions. It’s worth noting that retiring yellow-label and old cars ranked behind phasing out outdated industrial capacity in PRD. FW replaced the PRD region as one of the three key air quality control regions since 2018, and the co-benefits of CO_2_ emission reduction in FW were larger from 2018 to 2020 than those from 2013 to 2017. The ranking of the measure-specific contributions in FW exhibited similarities with that in BTH, but promoting clean fuel in the residential sector was the largest contributor in FW. Clean air action-induced electricity consumption in BTH, YRD, and PRD led to increases in carbon emissions in the other regions that provide electricity to these three regions, which are estimated to be 7.9, 1.4, and 0.2 Mt CO_2_ in 2020 respectively (methods are shown in Supplementary Note 2 and Supplementary Table 4). Such emission increases are smaller than the local CO_2_ emission reductions in these three key regions and the co-benefits are still significant.

Figure 3e–f further presents CO_2_ emission reduction co-benefits by province. In general, provinces with stricter pollution control policies tend to obtain higher reductions in both ambient PM_2.5_ concentration and CO_2_ emissions. Hebei, Shandong, Zhejiang, Shanxi, and Henan attained the highest CO_2_ emission reduction due to the above co-beneficial measures, reducing local CO_2_ emissions by 5.9% to 13.2% in 2020. Hebei and Shandong are provinces with heavy industries (e.g., iron and steel) and massive coal use in both the industrial and residential sectors. They revealed an evident policy effectiveness (i.e., policy intensity, Supplementary Note 1) of control measures and a dramatic decrease in PM_2.5_ pollution, indicating that the adjustment of the industrial structure largely contributed to the attained co-benefits. In contrast, Tibet and Hainan did not implement stringent measures because they exhibit the best air quality in China, hence attaining the smallest reductions in CO_2_ emissions among all the provinces. These results suggest that controlling air pollution is a motivational factor in reducing provincial CO_2_ emissions from 2013 to 2020.

## Discussion

China has aggressively targeted air quality improvement through a series of measures since 2013. We found that, although the initial goal of China’s stringent clean air targets focused solely on the air pollution caused by short-lived reactive species, the stringent air quality target of China also functioned as a strong motivating force to simultaneously transform energy systems and mitigate CO_2_ emissions. Carbon-intensive infrastructures that persisted for a long time were rapidly phased out, and clean and energy-efficient factories survived in response to the implemented clean air measures. The average energy efficiency in China was thus promoted^31^, and the transition in China’s energy and industrial systems was accelerated.

China’s clean air policy has direct effects on the global carbon budget, and the effects continue to grow rapidly since China has dominated the global trend since 2010 and contributed 31% of the global fossil CO_2_ emissions in 2020^32^. China’s clean air actions drove a plateau in its CO_2_ emissions after 2013. This plateau in China’s emissions, combined with the emission reductions in the US and EU, have counterbalanced the emissions growth that occurred in India and in the rest of the world, which is the primary driving force that pulls global CO_2_ emissions off track and reaches a contemporary plateau over this period^33^.

Although the clean air measures implemented since 2013 have yielded remarkable achievements on CO_2_ emission reduction in China, continuous efforts are needed to optimize the country’s energy system and economic structure to promote green recovery after the COVID-19 pandemic as well as further encourage green growth in the future. After the 2013–2016 emission plateau, China’s CO_2_ emissions continued to grow from 2017 to 2019, reaching a new record high even larger than the 2013 emissions^9,32,34^, although the effort controlling air pollution continued. After the five-year clean air action, the energy efficiency of China’s industries has improved to a high level, with massive of the inefficient factories upgraded or phased out. Common solutions for climate change and air pollution are urgently needed, as China required all cities to meet current air quality standards before 2035^35,36^ and pledged to realize carbon neutrality before 2060^37^.

The average unit cost of per abated CO_2_ emission of five co-beneficial measures is estimated to be 95.6 $ ton^−1^ (methods and data are shown in Supplementary Note 3 and Supplementary Tables 5 and 6), higher than traditional CO_2_ mitigation measures such as the deployment of renewable energy (11.0–12.0 $ ton^−1^)^38^ and power plant technology upgrades (58.9 $ ton^−1^)^39^. However, the economic and health benefits of air quality improvements (e.g., avoided premature deaths) compensate for or even offset such large abatement costs^4,40^, which makes the CO_2_ co-benefits more attractive. The end-of-pipe control measures are effective options that improve the air quality in the short term, while the shrinking potential of end-of-pipe control^1^ may not be able to support the ambitious air quality goals in China^4^. A co-beneficial strategy should be adopted in future policy design to coordinate clean air measures and address climate change measures in different aspects, while measures yielding co-benefits should be prioritized. In addition to phasing out outdated capacity and eliminating scattered coal use, the development of renewable energy has also been accelerated since 2005 due to the Renewable Energy Act and contributed to considerable CO_2_ emission reduction^41,42^. According to our estimation (Supplementary Note 4), China’s accumulated CO_2_ emissions between 2013 and 2020 could be 3.78 Gt CO_2_ higher than the actual emission state if the increased electricity generation from renewable energy from 2013 to 2020 was fulfilled by thermal power plants. The development of renewables was excluded from our co-benefit analysis because it was not recognized as the consequence of the clean air action (Supplementary Table 7). However, the replacement of fossil fuel use with renewable capacities is indispensable to reach CO_2_ emission peaks and carbon neutrality in China.

China’s experiences with air pollution control while achieving CO_2_ emission reduction co-benefits have broad implications for other developing countries, such as countries in South Asia and Africa, which host the most air-polluted cities worldwide. These countries rely heavily on fossil fuel energy and emit massive air pollutants and greenhouse gases due to their soaring economic growth and abundant fossil fuel use. Since air pollution has strong effects on public health, government tends to address the worsening air quality as a top policy priority. China’s co-beneficial measures may provide feasible mitigation options in the near term for other developing countries facing air pollution problems, as a sharp transformation toward low-carbon growth may not be realistic for these developing countries because of financial pressure and other factors^43^.

## Methods

### CO_2_ emission estimates

The historical trends of CO_2_ emissions in China from 2005 to 2020 were estimated through a bottom-up approach with the MEIC model. The MEIC model(http://www.meicmodel.org) is a dynamic technology-based inventory model developed for China by Tsinghua University^2,18–22^, including the unified source categorization, emission factor database, technology-based method, and high-resolution emission processing system on the cloud computing platform. This study estimated CO_2_ emissions originating from fossil fuel combustion and cement production by multiplying activity data by corresponding emission factors:

1 $${E}_{i,j}={A}_{i,j}\times {{{{{{\rm{EF}}}}}}}_{i,j}$$

where *E*_*i,j*_ denotes the CO_2_ emissions of fossil fuel/industrial product *i* consumed or produced in sector *j*, *A*_*i,j*_ denotes the corresponding fuel consumption/industrial production provided by MEIC, and EF_*i,j*_ denotes CO_2_ emission factors obtained from Liu et al.^44^. Supplementary Fig. 2 compares CO_2_ emission estimated by this study with various data sources.

### Estimates of CO_2_ emission reduction from five co-beneficial measures

This work carried out an ex-post assessment of the CO_2_ emission reduction co-benefits of clean air measures in China from 2013 to 2020, based on the real implementation rate of each measure collected by the government afterwards. China’s top-down system used the engineering-oriented approach to set air quality targets and prescribe measures to reach the targets. The government inspected the actual progress of the measures regularly to ensure the prescribed measures were effectively implemented, and progress were summarized in statistical reports. This work collected the real implementation rate of clean air measures from provincial self-inspection reports, official news and other investigation reports. Combining the real implementation rate with the MEIC model and the Ministry of Ecology and Environment (MEP) database^45^, the CO_2_ emission reduction co-benefits were estimated.

Here we make a more detailed explanation of selected co-beneficial measures in our assessment. Five co-beneficial measures were summarized from Air Pollution Prevention and Control Action Plan^46^, Three-Year Action Plan for Winning the Blue Sky Defense Battle^47^, and regional action plans released to address the air pollution in autumn and winter (e.g., Action Plan to Comprehensive Control Autumn and Winter Air Pollution in Beijing-Tianjin-Hebei and Surrounding Regions 2017–2018^48^ and Action Plan to Comprehensive Control Autumn and Winter Air Pollution in Beijing–Tianjin–Hebei Areas and Fenwei Plain 2020–2021^49^). As shown in Supplementary Table 8, only measures implemented for the first time or strengthened (e.g., expanding the scope of management) since 2013 were selected (marked by green and blue shades).

The CO_2_ emission reduction co-benefits were estimated with Eq. (2).

2 $$\varDelta {E}_{k}=\mathop{\sum}\limits_{i}(\varDelta {{{{{\rm{A}}}}}}{1}_{i}\times {{{{{{\rm{EF}}}}}}}_{i})-\mathop{\sum}\limits_{j}(\varDelta {{{{{\rm{A}}}}}}{2}_{j}\times {{{{{{\rm{EF}}}}}}}_{j})$$

where ∆*E*_*k*_ denotes the co-benefits of CO_2_ emission reduction from measure *k*, *i* denotes the reduced fossil fuel/industrial product, *j* denotes the increased fossil fuel, and ΔA1 and ΔA2 denote the energy/industrial production reduction and the energy increase, respectively, due to measure *k*. For example, if a coal-fired boiler was replaced by an NG-fired boiler, ΔA1 denotes the annual coal use of the coal-fired boiler, and ΔA2 denotes the annual NG use of the new boiler. EF_*i*_ was retrieved from Liu et al.^44^. The measure-specific approaches for energy flow estimation are introduced below.

### (a) Upgrades on industrial boilers

Small, polluting coal-fired industrial boilers were replaced by larger boilers or shifted to cleaner energy sources, leading to energy savings attributed to energy efficiency improvement (as indicated in Supplementary Table 9). The eliminated capacities of coal-fired boilers were collected from local self-inspection reports. The coal intensity was assumed as 366 tons coal per MW and 377 tons coal per MW for coal-fired industrial boilers and heating boilers, respectively, according to the estimation of the Beijing Clean Air Action Plan. Of the 424 GW of coal-fired boilers eliminated between 2013 and 2020, 192 GW was completely eliminated, 95 GW was replaced by larger boilers (central heating), and 112 GW was shifted to NG (Supplementary Table 10). The transformation from coal to electricity and biomass fuels was 5.9 GW and 18.5 GW, respectively. 63.6 Mtce coal has been saved by eliminating small coal-fired boilers in 2020.

### (b) Phasing out small and polluting factories

Since implementing end-of-pipe pollution control in small and polluting factories was neither practical nor cost-effective, tremendous effort was made to eliminate polluting small factories typically comprising super-emitters. The involved sector includes lime production, brick production, and other industrial processes. These small factories were assumed to be shut down completely, and the coal intensity values of the different products were collected from relevant standards and the Ministry of Ecology and Environment (MEP) database^45^. Phasing out small industrial furnaces (lime and brick furnaces) played a dominant role, reducing coal use by 22 Mtce in 2020. Coal reduction through the elimination of foundries, nonferrous metal factories, and other non-key industrial processes was estimated based on the reduced production and coal intensity, contributing a reduction of 4.0 Mtce in coal use in 2020 (Supplementary Table 11).

### (c) Phasing out outdated industrial capacity

Here, we mainly considered phasing out outdated industrial capacities in four key sectors: coal-fired power plants, iron and steel production (including coking), cement production, and glass production. Outdated capacities were assumed to be replaced by advanced capacities, and energy savings were estimated by the amount of eliminated outdated industrial capacities (provided by local self-inspection reports) and the difference in energy intensity between advanced and outdated technologies. Differences in the energy intensity were collected from the MEP database^45^. In 2020, phasing out small coal-fired power plants reduced coal use by 25.5 Mtce, while phasing out outdated capacities in the iron and steel production, cement production and glass production sectors reduced coal use by 31.1, 14.3, and 0.19 Mtce, respectively.

### (d) Promoting clean fuels in the residential sector

The co-reduction benefits of scattered coal use substitution were estimated by the number of households that replaced coal with cleaner energy, as reported by local governments. According to local self-inspection reports, among 12.7 million rural families involved in scattered coal substitution from 2013 to 2020, 54% of all households switched from coal to NG, 33% switched to electricity, 6% shifted to cleaner coal use, 5% switched to heating, and 1% eliminated coal use. The coal savings originating from scattered coal substitution in rural areas reached 24.7 Mtce in 2020. Scattered coal consumption per household was estimated based on the mean daily coal consumption per household and the central heating duration announced by regional governments (Supplementary Table 12)^50,51^. The energy efficiencies of different energy are listed in Supplementary Table 13. Biomass fuels are carbon neutral and were therefore not included in our estimates. In addition, the total elimination of coal-fired residential boilers reached 198 GW (Supplementary Table 14), reducing coal consumption by 29.5 Mtce in 2020. This part of calculation method was similar to that of upgrades on industrial boilers.

### (e) Retiring yellow-label and old vehicles

This measure corresponds to the strengthening of vehicle emission standards, as reported by Zhang^1^, retiring over 26.8 million yellow-label and old vehicles from 2013 to 2020. The number of eliminated yellow-label and old vehicles and other parameters were considered to estimate the resultant energy savings:

3 $${E}_{l}={{{{{\rm{VP}}}}}}\times {X}_{l}\times {{{{{{\rm{FE}}}}}}}_{l}\times {{{{{{\rm{VKT}}}}}}}_{l}$$

Where *l* is the vehicle types, VP is the number of eliminated yellow-label vehicles, *X*_*l*_ is the share of vehicle type *l*, FE_*l*_ is the fuel economy of vehicle type *l*, and VKT is the average vehicle mileage of vehicle type *l*. *X*, FE, and VKT were estimated with a vehicle emission model^20^. As indicated in Supplementary Table 15, the energy savings achieved with this measure reached 25.2 Mtce oil in 2020.

### Estimates of CO_2_ emission increase from strengthening industrial emission standards

Besides CO_2_ emission reduction due to energy-saving or energy transformation measures, this work also takes CO_2_ emission increase due to strengthening industrial emission standards into consideration. Previous work has demonstrated that this measure was significantly strengthened since 2013^1^. Here, we estimated increased CO_2_ emission from the wider application of end-of-pipe technologies in four key sectors: coal-fired power plants, iron and steel production, cement production, and industrial boilers.

The method of estimating direct and indirect CO_2_ emission increase follows Zhao’s work^52^. Direct CO_2_ emissions are produced through chemical reactions, which can be estimated from air pollutants emission reduction and chemical equations. Choosing SO_2_ elimination as an example, direct CO_2_ emissions are produced through reactions between limestone or slaked lime and SO_2_. Indirect CO_2_ emissions are produced due to extra electricity consumption, which were estimated based on the increased capacities attributed to end-of-pipe technologies and the intensity of electricity consumption. Note that due to the similar electricity intensity of various particulate matter (PM) control technologies, the additional CO_2_ emissions stemming from upgrades to PM control technologies (for example, the shift from electrostatic precipitators (ESP) to fabric filters (FAB) were not calculated. Setting 2012 as the base year, air pollutants emission reduction and increased capacities with end-of-pipe technologies were provided by MEIC. Supplementary Table 1 lists the estimates in the key sectors.

## Supplementary information

Supplementary information

Description of Additional Supplementary Information

Supplementary Data 1

## Data Availability

The data generated in this study are provided in the Supplementary Information. Data presented in all figures in the main text and the Supplementary Information are provided as Supplementary Dataset. The map used in Fig. 3e is generated from open-source data provided by Ministry of Natural Resources of the People’s Republic of China (http://bzdt.ch.mnr.gov.cn/).

## Code availability

The code developed for this study is available from the corresponding author on reasonable request.
