# Supplementary material for: Co-benefits of CO2 emission reduction from China’s clean air actions between 2013-2020
Source: Nat Commun. 2022 Aug 27;13:5061. doi: 10.1038/s41467-022-32656-8 (PMC9419635; doi:10.1038/s41467-022-32656-8)
Supplement: Supplementary file 1 — Supplementary information [file 41467_2022_32656_MOESM1_ESM.pdf]

Co-benefits of CO_2 emission reduction from China's clean air actions between 2013-2020

Qinren Shi[1,2], Bo Zheng[3], Yixuan Zheng[2], Dan Tong[2], Yang Liu[2], Hanchen Ma[2], Chaopeng Hong[3], Guannan Geng[1], Dabo Guan[2], Kebin He[1], Qiang Zhang[2]

[1]School of Environment, Tsinghua University, Beijing, China

[2]Department of Earth System Science, Tsinghua University, Beijing, China

[3]Institute of Environment and Ecology, Tsinghua Shenzhen International Graduate School, Tsinghua University, Shenzhen 518055, China

These authors contributed equally to this work: Qinren Shi, Bo Zheng.

Correspondence to: Q. Zhang (qiangzhang@tsinghua.edu.cn)

Supplementary Note 1. Evaluation framework for policy effectiveness of five co-beneficial measures

The policy assessment framework and metrics (Supplementary Table 2) are built based on the information on regional clean air policy implementation to evaluate policy effectiveness (i.e., policy intensity) of five co-beneficial measures in a specific region. The evaluation score of measure *i* in region *j* is denoted as $Score_{i,j}$, which can be derived individually from Supplementary Table 2. Higher scores indicate higher policy effectiveness. Then the scores of five measures are summed and normalized to a range of 0 (very weak) to 1(very strong). Normalized regional scores are shown in Supplementary Table 3.

Supplementary Note 2. Method of estimating action-induced electricity consumption in BTH, PRD and YRD and relative CO_2 emission increase in other regions

BTH, YRD and PRD belongs to Northern power grid, Eastern power grid and Southern power grid, respectively. These three regions are major input points of electricity and lead to carbon increase in the major output points (Supplementary Table 4)[1]. Electricity increases due to the clean air action in BTH, YRD and PRD reached 29.7, 13.5 and 2.1 thousand GWh in 2020. Provincial output, input and total electricity generation of provinces in key regions were collected from China energy statistical yearbook and China Electric Power Yearbook, and import ratio of electricity in key regions and CO_2 emission factor of each power grid were estimated, and CO_2 emission increase in other regions could be calculated using following equation:

$$E_i = I_i \times R_i \times \mathrm{EF}_{\mathrm{grid}_i} \quad (1)$$

Where E_i is CO_2 increase in other regions due to action-induced electricity consumption in region *i*; I_i is the action-induced increased electricity in region *i*; R_i is the import ratio of electricity in region *i*; and $\mathrm{EF}_{\mathrm{grid}_i}$ is the average CO_2 emission factor of power grid to which region *i* belongs, respectively.

Supplementary Note 3. Method of estimating cost of five co-beneficial measures

Annualized costs were used to estimate cost of five co-beneficial measures. For measures that only involve the elimination of old equipment (phasing out small and polluting factories and retirement of yellow-label vehicles), cost is calculated as:

$$C_i = \mathrm{RV}_{\mathrm{eliminated}} \tag{2}$$

$$\mathrm{RV}_{\mathrm{eliminated}} = \mathrm{RV}_{\mathrm{all}} \times \frac{d(1+d)^r}{(1\text{-}d)^r\text{-}1} \tag{3}$$

Where C_i is annualized cost in year i; $\mathrm{RV}_{\mathrm{eliminated}}$ is annualized residual value of the eliminated equipment; RV_{all} is residual value of the eliminated equipment; d is discount rate (10% in this study); r is residual lifetime of the eliminated equipment.

For measures that involve both elimination of old equipment and construction of new equipment (phasing out outdated capacities, upgrading industrial boilers and promoting clean fuels), cost further includes annualized capital cost of the new equipment and additional annual operational cost from the new equipment:

$$\mathrm{C}_i = \mathrm{RV}_{\mathrm{eliminated}} + \mathrm{INV}_{\mathrm{new}} + \mathrm{FOM}_{\mathrm{additional}} \tag{4}$$

$$\mathrm{INV}_{\mathrm{new}} = \mathrm{INV}_{\mathrm{all}} \times \frac{d(1+d)^t}{(1\text{-}d)^t\text{-}1} \tag{5}$$

$$\mathrm{FOM}_{\mathrm{additonal}} = \mathrm{FOM}_{\mathrm{new}} - \mathrm{FOM}_{\mathrm{eliminated}} \tag{6}$$

Where $\mathrm{INV}_{\mathrm{new}}$ is the annualized capital cost; $\mathrm{INV}_{\mathrm{all}}$ is the total capital cost; t is lifetime of new equipment; $\mathrm{FOM}_{\mathrm{additonal}}$ is the additional annual operational cost due to the upgrade of equipment; $\mathrm{FOM}_{\mathrm{new}}$ and $\mathrm{FOM}_{\mathrm{eliminated}}$ represents the annual operational cost of the new equipment and the eliminated equipment, respectively.

Supplementary Table 5 show the key parameters and data sources of five co-beneficial measures. Following the method in previous work[2], this study collected local subsidies to represent the residual value of the eliminated capacities and equipment. Other parameters were collected from literatures or database. Note that for promoting clean fuels in the residential sector $\mathrm{RV}_{\mathrm{eliminated}}$ was ignored. Costs were converted to dollars in 2020.

Costs of five co-beneficial measures between 2013-2020 reached 254.3 billion dollars. Phasing out outdated industrial capacities is the largest contributor (40.9%), followed by upgrades on industrial boilers (28.8%) and retiring yellow-label and old vehicles

(15.0%). This result is comparable but lower than previous study[3], which may led by different assumptions of residual lifetime of eliminated equipment. As shown in Supplementary Table 6, the overall unit cost of per abated CO_2 emission during 2013-2020 is 95.6 dollars per metric ton of CO_2 abated, while measure-specific costs vary from 39.9-138.3 \$ ton^{-1}. These air pollution control measures could be more costly for every ton of CO_2 abated compared to other low-carbon measures, like updating traditional power plants (58.9 \$ ton^{-1})[4] or raising renewable energy ratio in power industry (11.0-12.0 \$ ton^{-1})[5].

Supplementary Note 4. Method of estimating CO_2 emission reduction from the development of renewables during 2013 to 2020

Here we majorly consider the impact from the development of renewable in power sector. This study developed a counterfactual scenario assuming that non-fossil electricity generation fixes at 2012 level, while total electricity generation and the energy intensity of thermal power generation remain the same with the reality. In other words, the counterfactual scenario assumes that the increased electricity generation from renewable energy from 2013 to 2020 was fulfilled by thermal power plants instead, and CO_2 emission reduction could be calculated through comparing counterfactual scenario and reality. Provincial electricity generation of various technologies (thermal, hydro, solar, wind and others) was collected from China Electric Power Yearbook, and energy consumption in power sector were provided by MEIC.

Supplementary Table 1. Increased CO_2 emission from wider application of end-of-pipe technologies in 2020

Sector	Direct emission (kilo-tons)	Indirect emission (kilo-tons)
Power	12.30	18.03
Iron and steel	1.14	14.64
Cement	0.86	0.06
Coal-fired boilers	0.73	0.05

Supplementary Table 2. The policy effectiveness metrics

First Tier judgement standards	Option	Second Tier judgement standards	Option	Score
1. The measure was published by the local governments.	Yes	Relative policy document set concrete targets (including scope and deadline).	Yes	2
			No	1
	No			0
2. The measure with concrete targets occurred before the clean air action (2013) in this region.	No			2
	Yes			0
3. The measure been implemented during the clean air action in this region.	Yes	The intensity of the implementation was stronger than that before the clean air action.	Yes	2
			No	1
	No			0
4. The achievements of the measure were reported by local self-reports or official website.	Yes	The achievements of the measure were quantified.	Yes	2
			No	1
	No			0
5.There were specific operation of the measure during the clean air action. (e.g., the campaign against heavy air pollution in autumn and winter in the Beijing-Tianjin-Hebei region and surrounding areas from 2017 to 2018 is a specific operation of eliminating scattered coal and phasing out small and polluted factories)	Yes			2
	No			0

Supplementary Table 3. Normalized regional scores

Regions	Phase out outdated industrial capacities	Upgrades on industrial boilers	Promote clean fuels in the residential sector	Phase out small and polluting factories	Retire yellow-label vehicles	Normalized score
Beijing	2	10	8	10	8	0.760
Tianjin	5.25	10	10	10	6	0.825
Hebei	5.25	10	10	10	6	0.825
Shanxi	5.25	10	10	10	6	0.825
Inner Mongolia	5.25	8	8	10	6	0.745
Liaoning	4	8	8	10	6	0.720
Jilin	3	8	6	10	6	0.660
Heilongjiang	4	8	7	10	6	0.700
Shanghai	3	8	0	10	6	0.54
Jiangsu	5.25	10	8	10	6	0.785
Zhejiang	5.25	10	0	10	6	0.625
Anhui	4.5	10	6	10	6	0.73
Fujian	4.25	8	0	10	6	0.565
Jiangxi	4.25	8	0	10	6	0.565
Shandong	5.25	10	10	10	6	0.825
Henan	5.25	10	10	10	6	0.825
Hubei	3.75	8	8	10	6	0.715
Hunan	4.25	8	0	10	6	0.565
Guangdong	5.25	10	0	10	6	0.625
Guangxi	4.25	8	0	10	6	0.565
Hainan	1.25	8	0	10	6	0.505
Chongqing	4.75	8	5	10	6	0.675
Sichuan	4.25	8	6	10	6	0.685
Guizhou	2.75	8	8	10	6	0.695
Yunnan	3.25	8	0	10	6	0.545
Tibet	2	8	0	10	6	0.520
Shaanxi	4.5	10	10	10	6	0.810
Gansu	4.25	8	8	10	6	0.725
Qinghai	4	8	8	10	6	0.720
Ningxia	4	8	8	10	6	0.720
Xinjiang	4	8	8	10	6	0.720

Supplementary Table 4. Major input and output points of Northern, Eastern and Southern power grid

Power grid	Major input points	Major output points
Northern power grid	Beijing, Tianjin and Hebei (BTH)	Shanxi, Inner Mongolia
Eastern power grid	Shanghai, Jiangsu and Zhejiang and Anhui (YRD)	Hubei, Anhui and Sichuan
Southern power grid	Guangdong (PRD)	Yunnan, Guizhou and Guangxi

Supplementary Table 5. Key parameters and data sources of five co-beneficial measures

Measures	Key parameters	Data Source
Phase out outdated industrial capacities	$RV_{eliminated}$	Local standard for special compensation funds
	r	MEP data
	INV_{new}	Zhang et al.[6]; Li et al.[7]; Wang et al.[8]
	FOM	
	t	Zhang et al.[9]; Tong et al.[10]
Promote clean fuels in the residential sector	INV_{new}	Liu et al.[2]
	FOM	
Upgrades on industrial boilers	$RV_{eliminated}$	Local subsidy standard for eliminating coal-fired boilers
	r	MEP database
	INV_{new}	Shen et al.[11]; Tang et al.[12]
	FOM	Zhang et al.[9]
	t	
Phase out small and polluting factories	$RV_{eliminated}$	Local standard for special compensation funds
	r	MEP database
Retire yellow-label vehicles	$RV_{eliminated}$	Local subsidy standard for eliminating yellow-label vehicles
	r	Zheng et al.[13]
	t	

Supplementary Table 6. Unit cost of per abated CO_2 emission of different measures

Measure	Unit cost \$ ton^{-1} abated CO_2	Data source
Average of all measures between 2013 and 2020	95.7	Estimated in this study
- Retire yellow-label and old vehicles	138.3	
- Phase out outdated industrial capacities	110.7	
- Upgrades on industrial boilers	92.4	
-Promote clean fuels in the residential sector	70.3	
- Phase out small and polluting factories	39.9	
Updating traditional power plants	58.9	Zhang et al.[4]
Raise renewable energy ratio in the power industry	11.0-12.0	Liang et al.[5]

Supplementary Table 7. Measures promoting renewables in the power sector from 2006 to 2020

2006-2010	2011-2012	2013-2017	2018-2020
Renewable Energy Act (REA)；　Compared with 2005, the proportion of nuclear power, hydropower and other renewable energy in primary energy production should increase by 0.1%, 0.8% and 0.4% respectively. （The 11th Five-year plan）	Develop hydropower in an orderly manner, develop nuclear power safely and efficiently, and accelerate the development of other renewable energy sources such as wind energy. Installed capacities of non-fossil power plants should reach 30% by 2015. The installed capacities of hydropower, wind power and solar power are supposed to reach 290, 100 and 21 GW by 2015.(The 12th Five-year plan)	Actively and orderly develop hydropower, develop and utilize geothermal energy, wind energy, solar energy and biomass energy. (The Action Plan)	Develop hydropower in an orderly manner and optimize the development layout of wind and solar energy. （Three-Year Action Plan）

Supplementary Table 8. Co-beneficial measures in China from 2006 to 2020.These measures are summarized from the following documents: Air Pollution Prevention and Control Action Plan[14], Three-Year Action Plan for Winning the Blue Sky Defense Battle[15], and regional action plans released to address the air pollution in autumn and winter (e.g., Action Plan to Comprehensive Control Autumn and Winter Air Pollution in Beijing-Tianjin-Hebei and Surrounding Regions 2017–2018[16] and Action Plan to Comprehensive Control Autumn and Winter Air Pollution in Beijing-Tianjin- Hebei Areas and Fenwei Plain 2020-2021[17]).

Measure	Scope	2006-2010	2011	2012	2013	2014	2015	2016	2017	2018	2019	2020
Phase out outdated capacities	Nationwide	Phase out outdated capacities (The 11th Five-Year Plan)	Phase out outdated capacities (The 12th Five-Year Plan)		Complete the task in the 12th Five-Year Plan one year ahead of schedule. (The Action Plan)		Eliminate outdated capacity with a wider scope and higher standards.(The Action Plan)			Increase efforts to eliminate outdated production capacity and reduce excess production capacity. Strictly prevent the production of low-quality steel. (Three-Year Action Plan)		
	Key regions									Promote the elimination of independent coking enterprises. Control the iron and steel production capacity of Hebei. (Three-Year Action Plan)		
Promote clean fuels in the residential sector	Key regions		Beijing: Use clean energy for residential heating(Beijing 12th Five-Year Plan)		Beijing: Complete the "coal-to-electricity" project of 44 thousand households in 2013. Promote scattered coal subsitution of the remaining 21 thousand households.(Beijing Action Plan)				Substitute coal by electricity and natural gas for over 3 million households in "2+26" cities. (Regional action plan 2017-2018)	Basically complete the replacement of residential scattered coal use in BTH and sorrounding areas and FW before the heating season in 2020. (Regional action plan 2020-2021)		
Upgrades on industrial boilers	Nationwide				Eliminate small coal-fired boilers (≤10 t/h capacity) in the built-up areas of cities at prefecture-level and above by 2017. Accelerate the construction of central heating, "coal-to-gas", and "coal-to-electricity" projects.(The Action Plan)					Basically eliminate small coal-fired boilers(≤10 t/h capacity) in the built-up areas of cities at county-level and above by 2020. (Three-Year Action Plan)		
	Key regions		Promote eliminating small coal-fired boilers(≤10 t/h capacity) in the built-up areas of cities at prefecture-level and above (The 12th Five-Year Plan)						Eliminate 44 thousand small coal-fired boilers on the task list. (Regional action plan 2017-2018)	Basically eliminate coal-fired boilers(≤35 t/h capacity) by 2020. (Three-Year Action Plan)		
Phase out small and polluting factories	Nationwide								Strengthen the comprehensive improvement of the environment of small enterprises. (Regional action plan 2017-2018)	Basically complete the task of phasing out small and polluting factories nationalwide by the end of 2020. (Three-Year Action Plan)		
	Key regions									BTH: Basically complete by the end of 2018. YRD and FW: Basically complete by the end of 2019. (Three-Year Action Plan)		
Retire yellow-label and old vehicles	Nationwide		Basically retire the yellow-label registered and operated before 2005 by 2015. (The 12th Five-Year Plan)		Basically retire the yellow-label car nationalwide by 2017. (The Action Plan)					Retire old vehicles. Promote eliminating diesel trucks that cannot meet the "China III" emission standard and old gas vehicles. (Three-Year Action Plan)		
	Key regions				Basically retire 5 million yellow-label vehicles in BTH, YRD and PRD by 2015.(The Action Plan)					Eliminate over 1 million medium-duty and heavy-duty diesel trucks that do not meet the "China III" emission standard in BTH and its surrounding areas and FW should by 2020. (Three-Year Action Plan)		

Note: Regional action plan represents for regional action plans released to address the air pollution in autumn and winter.

Supplementary Table 9. Assumption of energy efficiencies in industrial boiler sector

Type	Efficiency (%)
Small coal-fired boilers	65[18]
Larger coal-fired boilers/Central heating	84[19]
Electricity	98[20]
Natural gas	94[21]

Supplementary Table 10. Eliminated industrial coal-fired boilers between 2013-2020

Energy transformation type	Eliminated industrial coal-fired boilers (MW)							
	2013	2014	2015	2016	2017	2018	2019	2020
Completely eliminated	4492.7	16445.2	30707.2	38272.2	56507.9	16395.9	22831.4	6750.6
Larger coal-fired boilers/ Central heating	3189.5	10323.7	14289.6	21573.5	26458.1	8716.2	8158.5	2760.0
Electricity	79.8	566.8	941.1	1269.0	1723.0	682.7	438.3	219.6
Natural gas	12615.5	16055.3	17971.4	16562.5	29419.6	10465.4	5474.9	3078.9
Biomass fuel	175.2	754.6	4957.4	3956.3	4436.5	1382.6	2202.2	638.0
Total	20552.8	44145.6	68866.6	81633.6	118545.1	37642.9	39105.3	13447.1

Note: Origin unit of eliminated industrial coal-fired boilers is t h^{-1} in regional reports. 1 t h^{-1} = 0.65MW[22]. The numbers are annual progress compared to last year.

Supplementary Table 11. Coal use reduction of phasing out small and polluting factories

Product	Coal use reduction in 2020 (Mtce)
Lime	0.69
Brick	21.33
Foundry products	0.08
Electrolytic aluminum	0.79
Other non-ferrous metal	0.68
Paper products	2.43

Supplementary Table 12. Scattered coal consumption per household[23,24]

Region	Coal consumption per household (ton)
Beijing	3
Tianjin	2
Hebei	2.5
Shanxi	3.5
Inner Mongolia	3.7
Liaoning	3.1
Jilin	3.7
Heilongjiang	3.7
Jiangsu	2.3
Anhui	1.8
Shandong	2.5
Henan	2
Hubei	2.5
Sichuan	3.1
Guizhou	2.5
Tibet	2.5
Shaanxi	2.5
Gansu	3.7
Qinghai	3.7
Ningxia	3.1
Xinjiang	3.7

Supplementary Table 13. Assumption of energy efficiencies in residential sector[25]

Fuel	Efficiency (%)
Scattered coal	40
Briquettes	75
Heating	90
Electricity	90
Natural gas	90

Supplementary Table 14. Amount of eliminated residential coal-fired boilers between 2013-2020

	Eliminated residential coal-fired boilers (MW)							
Energy transformation type	2013	2014	2015	2016	2017	2018	2019	2020
Completely eliminated	2529.9	10033.4	12960.7	15122.8	30513.6	12284.1	9425.3	4315.5
Larger coal-fired boilers/Central heating	2616.7	10968.5	7690.2	10298.4	15833.1	7601.4	3963.0	1975.2
Electricity	43.2	1344.6	695.1	1783.6	1960.8	927.9	619.0	239.2
Natural gas	3185.4	2601.6	3698.4	3345.9	8183.8	3186.7	1611.8	847.4
Biomass fuel	80.5	233.7	1068.7	1328.4	947.7	610.4	858.8	162.8
Total	8455.6	25181.7	26113.1	31879.1	57439.0	24610.6	16477.9	7540.2

Note: Origin unit of eliminated industrial coal-fired boilers is t h^{-1} in regional reports. 1 t h^{-1} = 0.65MW[22]. The numbers are annual progress compared to last year.

Supplementary Table 15. Energy saving in the transportation sector between 2013-2020

Fuel (10^4 tce)	2013	2014	2015	2016	2017	2018	2019	2020
Diesel	63.6	329.8	312.3	245.5	198.5	131.9	104.1	86.2
Gasoline	38.1	197.6	187.1	147.1	118.9	219.5	73.8	61.1

Note: The numbers are annual progress compared to last year.

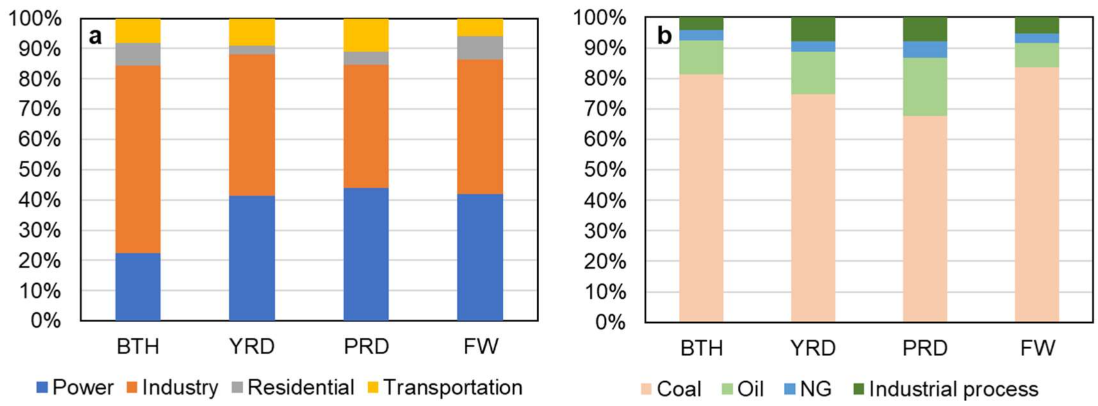

Supplementary Figure 1. CO_2 emission structures of four key regions in 2013. **A** shows sectoral results. **b** shows emission proportion of different types of fossil fuel combustion and industrial process (cement production).

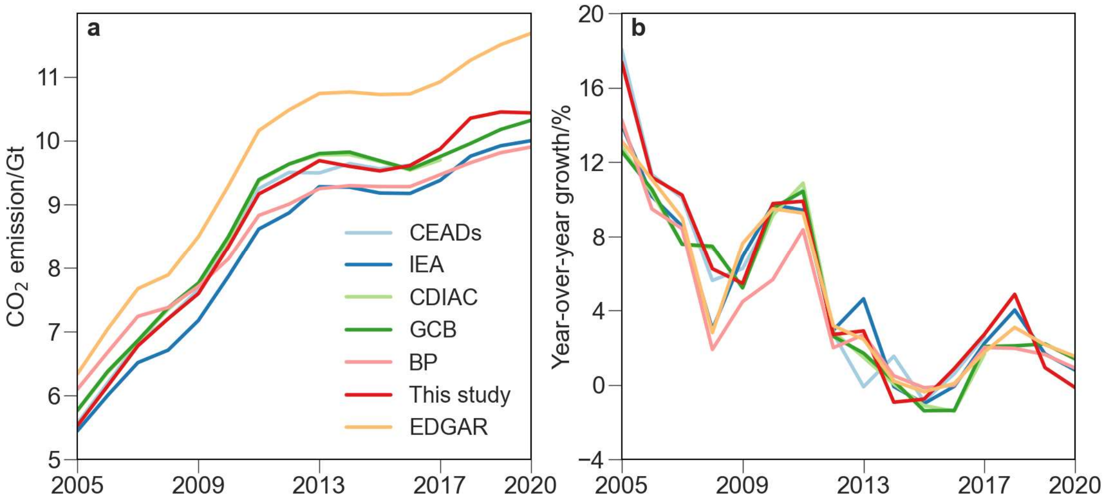

Supplementary Figure 2. Comparison of historical CO_2 emission and growth rates between different data source. Different curves represent various data sources, including IEA (dark blue curve, emissions from combustion of fossil fuels only)[26,27], BP (pink curve, emissions from combustion of fossil fuels only)[28], EDGAR (orange curve, emissions from fossil fuels combustion and processes)[29], CDIAC (light green curve, emissions from combustion of fossil fuels and cement production)[30], CEADs (light blue curve, emissions from combustion of fossil fuels and cement production)[31,32], and GCB (dark green curve, emissions from combustion of fossil fuels and cement production)[33]. The red curve shows the real emission estimates from this study, including emissions from fossil fuel combustion and cement production.

Supplementary References

1 Ma, L. & Zhang, B. The spatial distribution and evolution of inter provincial electricty flow in China. *Journal of Natural Resources* **34**, 348-358,(2019).

2 Liu, H. & Mauzerall, D. L. Costs of clean heating in China: Evidence from rural households in the Beijing-Tianjin-Hebei region. *Energy Economics* **90**,(2020).

3 Zhang, J. *et al.* Cost-benefit analysis of China's Action Plan for Air Pollution Prevention and Control. *Frontiers of Engineering Management* **6**, 524-537,(2019).

4 Zhang, Y., Wang, C., Wang, K. & Chen, J. CO_2 emission scenario analysis for China's electricity sector based on LEAP software. *Journal of Tsinghua University (Science and Technology)* **47**, 365-368,(2007).

5 Liang, Y., Yu, B. & Wang, L. Costs and benefits of renewable energy development in China's power industry. *Renewable Energy* **131**, 700-712,(2019).

6 Zhang, Z. *Cost-benefit Analysis of Fine Particulate Pollution Control in Shenzhen City* Master thesis, Tsinghua University, (2017).

7 Li, B. S. *et al.* Climate and Health Benefits of Phasing out Iron & Steel Production Capacity in China: Findings from the Imed Model. *Clim Chang Econ* **11**,(2020).

8 Wang, J. *Synergetic Control Strategy for Multiple Air Pollutants from Multiple Sectors in the Yangtze River Delta Region* Master thesis, Tsinghua University, (2014).

9 Zhang, F. *et al.* Estimation of abatement potentials and costs of air pollution emissions in China. *J Environ Manage* **260**, 110069,(2020).

10 Tong, D. *et al.* Current Emissions and Future Mitigation Pathways of Coal-Fired Power Plants in China from 2010 to 2030. *Environ Sci Technol* **52**, 12905-12914,(2018).

11 Shen, B., Han, Y., Price, L., Lu, H. & Liu, M. Techno-economic evaluation of strategies for addressing energy and environmental challenges of industrial boilers in China. *Energy* **118**, 526-533,(2017).

12 Tang, Y. *Research on Evaluation of Replacement Strategy for Coal-fired Boilers in Industrial Enterprises* Master thesis, Nanjing University of Aeronautics and Astronautics, (2020).

13 Zheng, B. *et al.* High-resolution mapping of vehicle emissions in China in 2008. *Atmospheric Chemistry and Physics* **14**, 9787-9805,(2014).

14 State Council of the People's Republic of China. Notice of the general office of the state council on issuing the air pollution prevention and control action plan. http://www.gov.cn/zwgk/2013-09/12/content_2486773.htm, Accessed 12 September 2013

15 State Council of the People's Republic of China. Notice of the state council on issuing the three-year action plan for winning the Blue Sky defense battle. http://www.gov.cn/zhengce/content/2018-07/03/content_5303158.htm, Accessed 27 June 2018

16 Ministry of Ecology and Environment of the People's Republic of China. Notice of the state council on issuing the action plan to comprehensive control autumn and winter air pollution in Beijing-Tianjin-Hebei and surrounding regions 2017–2018. https://www.mee.gov.cn/gkml/hbb/bwj/201708/t20170824_420330.htm, Accessed 21 August 2017

17 Ministry of Ecology and Environment of the People's Republic of China. Notice of the state council on issuing the action plan to comprehensive control autumn and winter air pollution in Beijing-Tianjin- Hebei areas and Fenwei Plain 2020-2021. https://www.mee.gov.cn/xxgk2018/xxgk/xxgk03/202011/t20201103_806152.html, Accessed 30 October 2020

18 Zhao, Y. *The Exploration Research to Carbon Emissions Problems on the Utilization of Central Heating Reconstruction Project*, (2015).
19 Special Equipment Safety Supervision Bureau of AQSIQ. *Supervision Regulation on Energy Conservation Technology for Boiler*, (2010).
20 He, R., Meng, X., Li, Y., deng, D. & Liao, H. Analysis of Electric Boiler Technology and Environmental Effect of Its Application. *Environmental Science and Management* **042**, 102-105,(2017).
21 Zhang, G. *et al.* Evaluation and Benefit Analysis of Small Coal-fired Boiler Alternative Program: A Case Study of Jinan City *Environmental Protection Science* **043**, 79-84,(2017).
22 Guo, Y., Tian, J. P., Chertow, M. & Chen, L. J. Exploring Greenhouse Gas-Mitigation Strategies in Chinese Eco-Industrial Parks by Targeting Energy Infrastructure Stocks. *J Ind Ecol* **22**, 106-120,(2018).
23 Chen, H. & Chen, W. Potential impact of shifting coal to gas and electricity for building sectors in 28 major northern cities of China. *Applied Energy* **236**, 1049-1061,(2019).
24 Zhu, X. *et al.* Stacked Use and Transition Trends of Rural Household Energy in Mainland China. *Environmental Science & Technology* **53**, 521-529,(2019).
25 Wu, J., Cheng, L., Lu, Y. & Song, L. Life cycle inventory analysis of clean alternatives to scattered coal heating. *Environmental Science* **v.38**, 372-380,(2018).
26 The International Energy Agency. Greenhouse Gas Emissions from Energy: Overview. (2021).
27 The International Energy Agency. *After steep drop in early 2020, global carbon dioxide emissions have rebounded strongly*, https://www.iea.org/news/after-steep-drop-in-early-2020-global-carbon-dioxide-emissions-have-rebounded-strongly (2021).
28 British Petroleum. Statistical Review of World Energy 2021. (2022).
29 Crippa, M. *et al.* GHG emissions of all world countries - 2021 Report. Report No. 978-92-76-41547-3, (Publications Office of the European Union, 2021).
30 Gilfillan D, M., G, Boden T, and Andres R. Global, Regional, and National Fossil-Fuel CO_2 Emissions: 1751-2017 CDIAC-FF. *Research Institute for Environment, Energy, and Economics, Appalachian State University*,(2020).
31 Shan, Y. *et al.* China CO_2 emission accounts 1997-2015. *Sci Data* **5**, 170201,(2018).
32 Shan, Y., Huang, Q., Guan, D. & Hubacek, K. China CO_2 emission accounts 2016-2017. *Sci Data* **7**, 54,(2020).
33 Friedlingstein, P. *et al.* Global Carbon Budget 2021. *Earth Syst. Sci. Data* **14**, 1917-2005,(2022).
