# Supplementary material for: Co-benefits of CO2 emission reduction from China’s clean air actions between 2013-2020
Source: Nat Commun. 2022 Aug 27;13:5061. doi: 10.1038/s41467-022-32656-8 (PMC9419635; doi:10.1038/s41467-022-32656-8)
Supplement: Supplementary file 2 — Description of Additional Supplementary Information [file 41467_2022_32656_MOESM2_ESM.docx]

**Inventory of Supporting Information**

- Supplementary Information.
- Supplementary Data 1: Contains data presented in all figures in the main text and the Supplementary Information.
